# Predictive value of chemotherapy-related high-density lipoprotein cholesterol (HDL) elevation in patients with colorectal cancer receiving adjuvant chemotherapy: an exploratory analysis of 851 cases

**DOI:** 10.18632/oncotarget.10145

**Published:** 2016-06-17

**Authors:** Yun Wang, Zhi-qiang Wang, Feng-hua Wang, Xue-fen Lei, Shu-mei Yan, De-shen Wang, Fei Zhang, Rui-hua Xu, Ling-yun Wang, Yu-hong Li

**Affiliations:** ^1^ Sate Key Laboratory of Oncology in South China, Sun Yat-sen University Cancer Center, Collaborative Innovation Center for Cancer Medicine, Guangzhou, P.R. China; ^2^ Department of Medical Oncology, Sun Yat-sen University Cancer Center, Guangzhou, P.R. China; ^3^ Department of Medical Oncology, The Second Affiliated Hospital of Kunming Medical University, Kunming, P.R. China; ^4^ Department of Pathology, Sun Yat-sen University Cancer Center, Guangzhou, P.R. China; ^5^ Department of Gastroenterology, Sun Yat-sen Memorial Hospital, Sun Yat-sen University, Guangzhou, P.R. China

**Keywords:** colorectal cancer, high-density lipoprotein cholesterol, chemotherapy, prognosis

## Abstract

**Background:**

The phenomenon of chemotherapy-related lipid alterations has been reported based on a small number of patients and varies among different cancers. However, little is known about these alterations in colorectal cancer (CRC) patients.

**Results:**

Patients in cohort 1, but not in cohort 2, exhibited significantly increased cholesterol, triglyceride, HDL-C, and ApoA-I levels, and decreased LDL-C and ApoB levels after adjuvant chemotherapy. Patients with chemotherapy-related HDL-C elevation exhibited better 3-year DFS (84.5% *vs*. 73%, *P* = 0.001) and 7-year OS (82% *vs*. 70%, *P* = 0.002) than those without. Similarly, the 3-year DFS (83.3% *vs*. 77.6%, *P* = 0.008) and 7-year OS (81% *vs*. 74.6%, *P* = 0.040) were superior in chemotherapy-related ApoA-I elevation patients. However, only HDL-C elevation remained an independent prognostic value in the multivariate Cox model.

**Methods:**

Eight hundred fifty-one CRC patients with curative-intent resection were retrospectively analyzed. Six hundred sixty-seven receiving fluoropyrimidine-based adjuvant chemotherapy for more than 3 months were enrolled in cohort 1. The lipid alterations before and after chemotherapy were studied. Simultaneously, 184 patients not treated with chemotherapy (cohort 2) were included as a control for the comparisons of lipids alterations within 1 month after resection and at half-year follow-up. Furthermore, these significant alterations were investigated with respect to the prognostic value of disease-free survival (DFS) and overall survival (OS). An internal validation was performed.

**Conclusion:**

We observed significant changes in the levels of various lipids in CRC patients receiving adjuvant chemotherapy. Furthermore, chemotherapy-related HDL-C elevation was determined to be an independent prognostic indicator for superior DFS and OS.

## INTRODUCTION

Colorectal cancer (CRC) is the third most common malignancy and the fourth leading cause of cancer worldwide. During the past 10 years, the incidence of CRC in China has increased 4-6% annually, representing 376.3 and 191.0 thousand new cases and deaths in 2015, respectively [1, 2]. Surgical resection is the mainstay curative treatment modality. However, approximately 20-45% patients develop metastasis and/or recurrence disease [3, 4]. Therefore, fluoropyrimidine-based chemotherapy regimens are recommended as adjuvant treatment for patients with stage III or stage II with risk factors after curative-intent resection.

In the past, abundant evidence has suggested the important role of aberrant lipid metabolism in the pathogenesis and progression of cancers [5–7]. Recently, chemotherapy drugs were reported to affect lipid profiles. For instance, significant increases of serum total cholesterol and low-density lipoprotein cholesterol (LDL-C) were observed after effective chemotherapy in patients with malignant lymphoma, small-cell lung carcinoma and urothelial-cell carcinoma but not breast cancer [8]. In metastatic CRC patients, decreased LDL-C and cholesterol and increased high-density lipoprotein cholesterol (HDL-C) were observed after anti-angiogenic combined chemotherapy [9]. However, these reports were based on a small number of patients and varied among different cancer types.

It remains unclear whether lipid alterations exist in CRC patients receiving fluoropyrimidine-based adjuvant chemotherapy. Furthermore, given the association between aberrant lipid metabolism and the pathogenesis and progression of cancers whether chemotherapy-related lipid alterations play roles in the prognosis of CRC is also speculative. Based on this knowledge, we conducted this retrospective study in CRC patients after radical resection to investigate the fluctuations of lipid profiles after the fluoropyrimidine-based adjuvant chemotherapy and assessed its roles in prognosis.

## RESULTS

### Patient characteristics

Table 1 presents the baseline clinicopathological characteristics of cohort 1 and 2. Cohort 1 included 667 CRC patients who underwent fluoropyrimidine-based adjuvant chemotherapy, whereas cohort 2 consisted of 184 contemporary patients not undergoing chemotherapy. The median age was 55 years in cohort 1 but was relatively older in cohort 2 (Median: 62.5 years). The population in both cohorts was predominantly comprised of men. The distribution of TNM stages was approximately equally distributed between stage II (42.9%) and III (57.1%) in cohort 1 but was predominated by stage II (80.1%) in cohort 2. The chemotherapy regimens administered in cohort 1 consisted of CapeOx (61.8%, 412 patients), FOLFOX (24.1%, 161 patients), capecitabine (10.6%, 71 patients), 5- fluorouracil (FU)/leucovorin (LV) (2.4%, 16 patients) and other regimens including fluoropyrimidine-agents (1.0%, 7 patients). 5-Fu/LV or capecitabine was predominantly delivered to stage II patients (86.2%, 72/87 patients), while FOLFOX or CapeOx was mainly administrated in those with stage III disease (63.7%, 365/573 patients). All patients in cohort 1 had received adjuvant chemotherapy, and 115 (17.2%) of the patients with rectal cancer also had received pre- or post-operative radiotherapy. One hundred eleven patients (16.6%) received concurrent chemotherapy, including CapeOx (82.9%), capecitabine (2.7%), FOLFOX (12.6%), or S-1(1.8%).

**Table 1:** Clinicopathological characteristics of included study cohorts (cohort 1: *N* = 667; cohort 2: *N* = 184)

Characteristics and lipid fluctuation		Cohort 1 (*N* = 667)	Cohort 2 (*N* = 184)	*P* value
		*N* (%)	*N* (%)	
Age at diagnosis	Median (range)	55 (23-75)	62.5 (20-82)	
	≤ 65 year	566 (84.9)	106 (57.6)	< 0.001
	> 65 year	101 (15.1)	78 (42.4)	
Gender	Male	417 (62.5)	110 (59.8)	0.499
	Female	250 (37.5)	74 (40.2)	
BMI	Median (range)	22.6 (13.5-34.3)	22.2 (14.8-34.9)	
	< 24 kg/m^2^	440 (66.0)	127 (29)	0.437
	≥ 24 kg/m^2^	227 (34.0)	57 (31)	
Location of primary tumor	Colon	340 (51.0)	101 (54.9)	0.347
	Rectum	327 (49.0)	83 (45.1)	
Histological subtype	Non-mucinous	609 (91.3)	168 (91.3)	1.000
	Mucinous	58 (8.7)	16 (8.7)	
Tumor grade	G1	148 (22.2)	32 (17.4)	0.158
	G2-3	519 (77.8)	152 (82.6)	
T-stage	pT1-3	138 (20.8)	30 (16.3)	0.186
	pT4	529 (79.3)	154 (83.7)	
N-stage	pN0	286 (42.9)	135 (73.4)	< 0.001
	pN1-2	381 (57.1)	49 (26.6)	
TNM-stage	Stage I	0 (0)	4 (2.2)	< 0.001
	Stage II	286 (42.9)	129 (80.1)	
	Stage III	381 (57.1)	51 (27.7)	
Tumor size	≤ 4 cm	396 (59.4)	99 (53.8)	0.175
	> 4 cm	271 (40.6)	85 (46.3)	
pre-operative CEA^§^	Positive	380 (57.0)	102 (55.4)	0.710
	Negative	287 (43.0)	82 (44.6)	
pre-operative CA19-9	≤ 30 cm	527 (79.0)	145 (78.8)	0.952
	> 30 cm	140 (21.0)	39 (21.2)	
Smoker	Yes	151 (22.6)	37 (20.1)	0.485
	No	516 (77.4)	146 (79.3)	
Dignosis of diabetes	Yes	37 (5.5)	4 (2.2)	0.060
	No	630 (94.5)	179 (97.3)	
Dignosis of hypertension	Yes	95 (14.2)	24 (13)	0.678
	No	572 (85.8)	160 (87)	
Radiotherapy	Yes	115 (17.2)		
	No	552 (82.8)		
Chmotherapy used	5-Fu+LV/capecitabine	87 (13.0)		
	FOLFOX/CapeOx	573 (85.9)		
	Others*	7 (1.0)		

BMI = Body Mass Index, CEA = carcinoembryonic antigen, CA19-9 = Carbohydrate antigen 19-9.

§ The reference value of CEA: nonsmoker ≤ 2.5 ng/ml, smoker ≤ 5ng/ml.

* Other regimens including fluoropyrimidine-agents.

### Lipid fluctuations and the predictive value

To measure the association between lipid fluctuations and chemotherapy, the paired *t*-test was applied to compare the difference in the lipid levels before and after chemotherapy in cohort 1, as well as within 1 month after resection and at the 6-month follow-up in cohort 2. Patients in cohort 1 exhibited the significantly increased cholesterol (*P* < 0.001), triglyceride (*P* < 0.001), HDL-C (*P* < 0.001), and ApoA-I levels (*P* < 0.001) and decreased LDL-C (*P* < 0.001) and apolipoprotein B (ApoB) levels (*P* = 0.004) after adjuvant chemotherapy. No significant lipid alterations were observed in patients without adjuvant chemotherapy in cohort 2 (Table 2). In addtion, to investigate the potential effect of the age, tumor stage, grade, gender, body mass index (BMI) and different regimens on chemotherapy-related lipid fluctuations, a stratified analysis was also performed in cohort 1. Even though the chemotherapy-related lipid alterations were inconsistent between various subgroups, HDL-C remained to increase after chemotherapy in every subgroup significantly (Stable 1).

**Table 2:** The alterations of lipids in cohort 1 and cohort 2

	Cohort 1 (*N* = 667)				Cohort 2 (*N* = 184)			
	Pre-CT	Post-CT	Difference^ƪ^	P value^ф^	Level at diagnoses	Level at follow-up	Difference&	*P* value^ф^
Cholesterol (mmol/L)	4.91±0.97	5.09±1.00	0.18±1.03	**< 0.001**	4.64±0.99	5.11±4.48	0.46±4.56	0.169
Triglyceride (mmol/L)	1.41±0.88	1.70±1.01	0.29±1.08	**< 0.001**	1.52±1.16	1.52±1.05	0.00±1.35	0.986
HDL-C (mmol/L)	1.15±0.30	1.37±0.37	0.22±0.36	**< 0.001**	1.13±0.32	1.11±0.43	−0.03±0.43	0.373
LDL-C (mmol/L)	3.11±0.86	2.96±0.90	−0.15±0.91	**< 0.001**	2.93±0.83	2.87±0.90	−0.06±0.98	0.380
ApoA-I (g/L)	1.18±0.37	1.36±0.28	0.18±0.43	**< 0.001**	1.13±0.24	1.12±0.30	−0.01±0.33	0.785
ApoB (g/L)	0.98±0.50	0.92±0.26	−0.06±0.49	**0.004**	1.04±1.05	0.96±0.77	−0.07±1.28	0.430

HDL-C = high-density lipoprotein cholesterol, LDL-C = low-density lipoprotein cholesterol, ApoA-I = apolipoprotein A-I, ApoB = apolipoprotein B, CT = chemotherapy. Data are mean ± standard deviations.

ƪ Difference = lipids_post-CT_ - lipids_pre-CT_. &Difference = lipids_follow-up._ -lipids_at diagnoses_

ф Compared with paired t-test.

**Figure 1:**
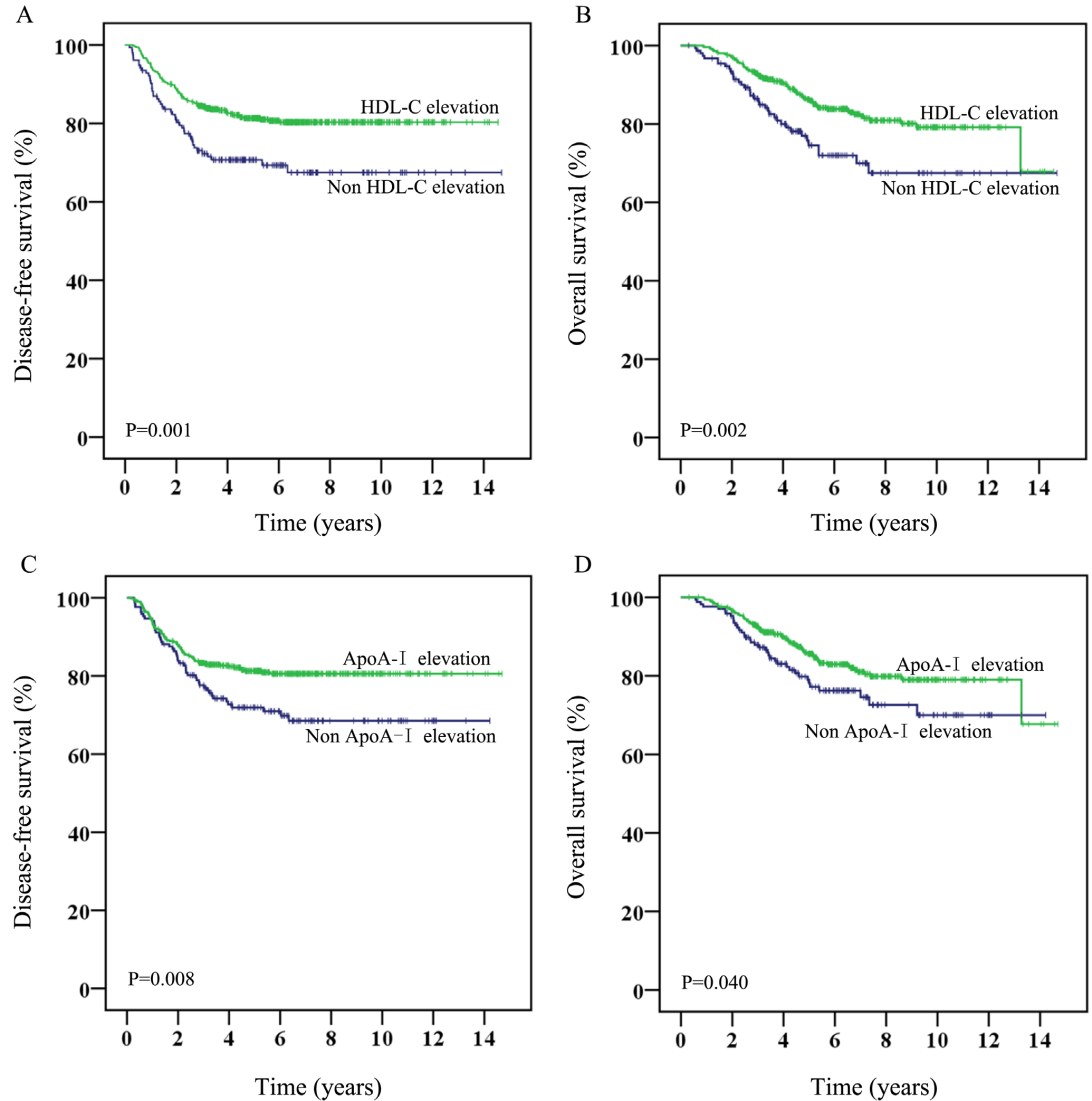
Survival of CRC patients receiving adjuvant chemotherapy stratified by chemotherapy-related HDL-C or ApoA-I alteration (cohort 1: *N* = 667) **A.** and **B.** Kaplan-Meier plots of disease-free survival and overall survival based on chemotherapy-related HDL-C alteration, respectively. **C.** and **D.** Kaplan-Meier curves of disease-free survival and overall survival based on chemotherapy-related ApoA-I alteration, respectively.

According to the comparison before and after chemotherapy in cohort 1, we further identified 379 (56.8%) patients with elevated cholesterol, 431 (64.6%) patients with elevated triglycerides, 512 (76.8%) patients with elevated HDL-C, 498 (74.7%) patients with elevated ApoA-I, 371 (55.6%) patients with decreased LDL-C, and 369 (55.3%) patients with decreased ApoB. We further investigated the prognostic value of various chemotherapy-related lipid fluctuations. The median follow-up was 5.5 years (IQR: 3.7-7.6 years). As shown in Table 3, chemotherapy-related HDL-C elevation and ApoA-I elevation exhibited significant associations with disease-free survival (DFS) and overall survival (OS). Patients with HDL-C elevation during chemotherapy exhibited better 3-year DFS (84.5% *vs*. 73%, *P* = 0.001, Figure 1A) and 7-year OS (82% *vs*. 70%, *P* = 0.002, Figure 1B) than those without HDL-C elevation. Similarly, the 3-year DFS and 7-year OS were superior in chemotherapy-related ApoA-I elevation patients (3-year DFS: 83.3% *vs*. 77.6%, *P* = 0.008, Figure 1C; 7-year OS: 81% *vs*. 74.6%, *P* = 0.040, Figure 1D; respectively).

**Table 3:** Chemotherapy-related lipids alteration for PFS and OS (cohort 1: *N* = 667)

Lipid fluctuations		*N* (%)	3-year DFS (%)	*P* value	7-year OS (%)	*P* value
Cholesterol elevation*	Yes	379 (56.8)	81.9	0.680	79.6	0.910
	No	288 (43.2)	81.9		79.0	
Triglyceride elevation*	Yes	431 (64.6)	81.1	0.398	79.5	0.824
	No	236 (35.4)	83.4		79.2	
HDL-C elevation*	Yes	512 (76.8)	84.5	0.001	82.0	0.002
	No	155 (23.2)	73.0		70.0	
LDL-C reduction*	Yes	371 (55.6)	80.8	0.203	79.0	0.665
	No	296 (44.4)	83.3		79.8	
ApoA-I elevation*	Yes	498 (74.7)	83.3	0.008	81.0	0.040
	No	169 (25.3)	77.6		74.6	
ApoB reduction*	Yes	369 (55.3)	82.0	0.938	78.3	0.669
	No	298 (44.7)	81.8		80.2	

* Comparision between before and after chemotherapy.

### Univariate and multivariate Cox analysis for DFS and OS

Furthermore, the prognostic value of HDL-C elevation after adjuvant chemotherapy was observed in univariate Cox regression analysis both for superior DFS (hazard ratio[HR]: 0.56; 95% confidence interval[CI]: 0.39-0.80, *P* = 0.001) and OS(HR[95%CI]: 0.54[0.37-0.80], *P* = 0.002) and was maintained in the multivariate Cox model (HR [95%CI] for DFS: 0.59[0.41-0.84], *P* = 0.004; HR[95%CI] for OS: 0.56[0.38-0.83], *P* = 0.004; Table 4). However, the positive predictive ability of ApoA-I elevation was only observed in univariate Cox analysis for DFS and OS but not in multivariate analysis (Table 4). Location of the primary tumor (colon cancer *vs*. rectal cancer) was also a superior independent indicator for DFS and OS. Other inferior independent indicators included advanced T-stage, N-stage and higher level preoperative CA19-9 for DFS, as well as older age, male sex, advanced N-stage, and higher level preoperative CA19-9 for OS (Table 4).

**Table 4:** Predictive factors for survival by univariate and multivariate analysis in cohort 1 (*N* = 667)

		Univariate analysis		Multivariate analysis	
		HR (95%CI)	*P* value	HR (95%CI)	*P* value
**Disease-free survival**					
Location of tumor	colon *vs*. rectal	0.68 (0.48-0.95)	**0.023**	0.67 (0.48-0.94)	**0.021**
T-stage	pT4 *vs*. pT1-3	1.88 (1.13-3.13)	**0.015**	1.99 (1.20-3.31)	**0.008**
N-stage	pN2 *vs*. pN0-1	2.54 (1.74-3.73)	**< 0.001**	2.49 (1.69-3.66)	**< 0.001**
Pre-operative CEA^§^	pos *vs*. neg	1.48 (1.05-2.10)	**0.026**	-	ns
Pre-operative CA19-9	> 30 *vs*. ≤ 30	1.62 (1.12-2.34)	**0.010**	1.52 (1.05-2.21)	**0.028**
HDL-C elevation	yes *vs*. no	0.56 (0.39-0.80)	**0.001**	0.59 (0.41-0.84)	**0.004**
ApoA-I elevation	yes *vs*. no	0.63 (0.44-0.89)	**0.008**	**-**	ns
**Overall survival**					
Age	> 65 *vs*. ≤ 65	1.79 (1.17-2.75)	**0.008**	1.82 (1.17-2.82)	**0.007**
Gender	male *vs*. female	1.61 (1.07-2.41)	**0.022**	1.72 (1.14-2.60)	**0.010**
Location of primary tumor	colon *vs*. rectal	0.57 (0.39-0.82)	**0.003**	0.55 (0.37-0.80)	**0.002**
Tumor grade	G2-3 *vs*. G1	0.68 (0.46-1.00)	**0.048**	-	ns
N-stage	pN2 *vs*. pN0-1	2.46 (1.62-3.75)	**< 0.001**	2.50 (1.64-3.83)	**< 0.001**
Pre-operative CEA^§^	pos *vs*. neg	1.50 (1.03-2.20)	**0.037**	-	ns
Pre-operative CA19-9	> 30 *vs*. ≤ 30	1.58 (1.06-2.35)	**0.026**	1.62 (1.08-2.43)	**0.020**
HDL-C elevation*	yes *vs*. no	0.54 (0.37-0.80)	**0.002**	0.56 (0.38-0.83)	**0.004**
ApoA-I elevation*	yes *vs*. no	0.67 (0.45-0.98)	**0.041**	-	ns

§ The reference value of CEA: nonsmoker ≤ 2.5 ng/ml, smoker ≤ 5ng/ml.

* Comparision between before and after chemotherapy. ns, not significant.

### Internal validation

Internal validation using a re-sampling procedure was performed to investigate the robustness of the multivariate Cox model. The replication rate of HDL-C elevation was 84% and 86% for DFS and OS, with a mean HR of 0.58 and 0.56 for DFS and OS, respectively (Stable 2).

### Stratification analysis by different regimens

Patients in cohort 1 were further categorized into two stratification groups according to different chemotherapy regimens to exclude the influence of oxalipaltin. Both in patients treated with 5-Fu+LV/capecitabine and FOLFOX/CapeOx, chemotherapy-related HDL-C elevation remained the superior predictive ability for both 3-year DFS (5-Fu+LV/capecitabine: 95.0% *vs*. 80.8%, *P* = 0.030; FOLFOX/CapeOx: 83.1% *vs*. 71.1%, *P* = 0.004; Table 5) and 7-year OS (5-Fu+LV/capecitabine: 90.5% *vs*. 72.7%, *P* = 0.025; FOLFOX/CAPEOX: 80.7% *vs*. 69.2%, *P* = 0.007; Table 5).

**Table 5:** The predictive value of chemotherapy-related HDL-C elevation in different regimens

		*N* (%)	3-year DFS (%)	*P* value	7-year OS (%)	*P* value
**FOLFOX/CapeOx**						
HDL-C elevation*	Yes	446 (77.8)	83.1	**0.004**	80.7	**0.007**
	No	127 (22.2)	71.1		69.8	
**5-Fu+LV/capecitabine**						
HDL-C elevation*	Yes	60 (69.0)	95.0	**0.030**	90.5	**0.025**
	No	27 (31.0)	80.8		72.7	

* Comparision between before and after chemotherapy.

### Lipid-related factors according to chemotherapy-related HDL-C elevation

To investigate the association between chemotherapy-related HDL-C elevation and other lipid-related features, patients who were overweight (BMI ≥ 24 kg/m^2^, according to Chinese standard criteria) and those with diabetes, hypertension, smoking addiction and other chemotherapy-related lipid fluctuations were analyzed according to their chemotherapy-related HDL-C elevation. As listed in Table 6, chemotherapy-related cholesterol elevation, ApoA-I elevation, LDL-C reduction, and ApoB reduction were associated with chemotherapy-related HDL-C elevation.

**Table 6:** The association between the HDL-C elevation and other lipids-related factors in cohort 1 (*N* = 667)

Variables		HDL-C elevation*		*P* value
		Yes	No	
BMI	< 24 kg/m2	332	108	0.266
	≥ 24 kg/m2	180	47	
Smoker	Yes	113	38	0.524
	No	399	117	
Dignosis of diabetes	Yes	30	7	0.522
	No	482	148	
Dignosis of hypertension	Yes	74	21	0.778
	No	438	134	
Cholesterol elevation*	Yes	334	45	**< 0.001**
	No	178	110	
Triglyceride elevation*	Yes	328	103	0.586
	No	184	52	
LDL-C reduction*	Yes	262	109	**< 0.001**
	No	250	46	
ApoA-I elevation*	Yes	438	60	**< 0.001**
	No	74	95	
ApoB reduction*	Yes	265	104	**0.001**
	No	247	51	

* Comparision between before and after chemotherapy.

## DISCUSSION

In this current study, we observed significant changes in various lipids after adjuvant chemotherapy in CRC, including significant increases in cholesterol, triglyceride, HDL-C, and ApoA-I levels and decreases in LDL-C and ApoB levels. Furthermore, HDL-C elevation was found to be an independent prognostic indicator for both superior DFS and OS. To our knowledge, the present study is a much larger one in comparison with those previously reported on chemotherapy-related lipid fluctuations in CRC, and the first one to investigate its potential prognostic role.

Studies focused on chemotherapy-induced lipid alterations are limited and have reported inconsistent results. For instance, Alexopoulos first reported a significant chemotherapy-induced increase in cholesterol and LDL-C in 39 patients with cancer, including lymphoma, small cell lung carcinoma and urothelial cell carcinomas. However, only triglycerides were observed to significantly increase in 18 breast cancer patients [8]. Results from another 12 breast cancer patients demonstrated a significant reduction in HDL-C and ApoA-I and an elevation in ApoB after the treatment of either doxorubicin and cyclophosphamide followed by paclitaxel or epirubicin, cyclophosphamide and 5-Fu followed by docetaxel [10]. Bassani reported an increase in serum triglycerides and an obvious reduction in HDL-C due to high dose bexarotene therapy in a case with cutaneous T-cell lymphoma [11]. In another study conducted by Melichar and his colleagues, 31 patients with metastatic CRC were observed to experience a reduction in cholesterol and LDL-C as well as an increase in HDL-C after treatment with bevacizumab, oxaliplatin, 5-fluorouracil and leucovorin [9]. Nevertheless, all of these observations were based on a small number of patients. In our current study, we measured the lipid fluctuations after adjuvant chemotherapy in 667 stage II-III CRC patients. The significant increases in cholesterol, triglyceride, HDL-C and ApoA-I levels with concurrent decreases in LDL-C and ApoB were observed after the fluoropyrimidine-based chemotherapy. To exclude the impact of other confounding factors, the lipid fluctuations of contemporary cohort 2 patients who did not undergo adjuvant chemotherapy were analyzed as controls. There were no significant lipid alterations in these cohort 2 patients, underlining the association between lipid alterations and chemotherapy administration.

To date, the underlying mechanism of chemotherapy-related lipid alterations remains unclear. Alexopoulos attributed the phenomena to a reversion of cancer-related aberrant lipid profiles after effective chemotherapy treatment [8]. However, the CRC patients enrolled in our study all underwent primary tumor radical resection. Therefore, we suggest the effect of chemotherapy drugs on lipid metabolism. Consistently, a previous study reported that animal models with no tumor burden treated with fluorouracil exhibited significant reductions in total cholesterol and triglyceride after drug administration, which supported the interference of 5- fluorouracil with lipid metabolism [12]. It had been demonstrated that chemotherapy agents like doxorubicin can decrease ATP binding cassette transporter A1 (ABCA1), a receptor of ApoA-I, *via* a downregulation of the peroxisomal proliferator activated receptor γ (PPARγ) and liver X receptor α (LXRα) transcription factors in liver cells directly, but no effect on ABCA1 was observed by cyclophosphamide or paclitaxel [10]. Therefore, the different trends of chemotherapy-related lipid alterations among the different reports is probably due to the complicated mechanisms that varied by different cancer types, animal species and drugs.

In our study, both HDL-C and ApoA-I exhibited a strong prognostic role in the univariate analysis. As the only athero-protective lipid, HDL-C is known for of its ability to transport excess cholesterol from the periphery to the liver for excretion. Moreover, HDL-C has been demonstrated to exhibit antioxidant, anti-inflammatory, anti-thrombotic within the last decade and exhibits an inverse association with cancers [13–16]. HDL-C was demonstrated to reduce the risk of cancer by 36% for every 10 mg/dl increase and is positively correlated with the prognosis of numerous cancers [13–16]. ApoA-I, the predominant protein component of HDL that is responsible for the assembly and function of HDL, was also found to consistently exhibit a protective effect in non-small cell lung cancer and nasopharyngeal carcinoma [17–19]. The result of the multivariate model in our study revealed that only HDL-C elevation remained an independent predictive variable, which is consistent with the close association between HDL-C and ApoA-I.

The mechanism underlying the association between chemotherapy-related elevated HDL-C/ApoA-I and superior survival remains speculative. The HDL-C/ApoA-I elevation may play a role in the inhibition of tumor metastasis. Patients with metastasis often exhibit with lower HDL-C than those without metastasis [20]. Consistently, recent preclinical studies indicated the potential inhibitory effect of HDL-C on the tumor-permissive microenvironment instead of a direct effect on tumor cells [21–23]. By suppressing myeloid-derived suppressor cell (MDSC) recruitment and MMP9, a matrix-degrading enzyme critical for pro-angiogenesis, HDL-C/ApoA-I may inhibit tumor angiogenesis, invasion and metastasis [23]. HDL-C/ApoA-I may also increase the conversion of tumor-associated macrophages (TAMs) from an M2-like pro-tumor phenotype to an M1-like anti-tumor phenotype [23, 24]. In addition, increasing ApoA-I or ApoA-I /HDL mimetics may effectively protect against tumor development and growth in mice model studies [23, 25, 26], and both the innate and adaptive immune responses are required for full anti-tumor activity [21, 23]. However, further investigations into the complicated mechanism underlying the anti-tumor function of HDL-C/ApoA-I elevation are required.

Although the larger cohort of the present study and the internal validation enhanced the confidence of our results, the study is nonetheless limited by its retrospective nature and restriction within one institution.

In conclusion, we observed significant changes in the levels of various lipids in patients with CRC receiving adjuvant chemotherapy after curative-intent resection, including significant increases in cholesterol, triglyceride, HDL-C, ApoA-I levels and decreases in LDL-C and ApoB levels. Furthermore, HDL-C elevation after adjuvant chemotherapy was found to be an independent prognostic indicator for both superior DFS and OS.

## MATERIALS AND METHODS

### Patients

CRC patients receiving curative-intent resection treatment at the Sun Yat-sen University Cancer Center, China, between 2002 and 2012 were retrospectively reviewed in this study. The inclusion criteria were as follows: (a) pathologically confirmed colorectal cancer; (b) underwent the curative-intent resection; (c) postoperative pathologically identified negative margins; (d) no evidence of distant metastasis at the initial diagnosis; and (e) adequate clinicopathological and lipid information for analysis. Patients who had received fluoropyrimidine-based adjuvant chemotherapy more than 3 months (more than 4 cycles for 3 weekly dosing schedules, or 6 cycles for two weekly schedules) were enrolled in cohort 1. Other contemporary eligible patients who received surgery alone were enrolled in cohort 2 as controls.

### Information collection

The clinicopathological information was reviewed from patient charts. Information at diagnoses including, age, sex, height, weight, tumor location, histological subtype, tumor grade, pre-operative CEA and CA19-9, alcohol consumption, smoking history and comorbidities were collected both for cohort 1 and cohort 2. TNM stage was reclassified according to the Union International Control Cancer (UICC) staging system (the 7th version). BMI was calculated by the following formula: weight (in kilograms)/height (in meters^2^). For cohort 1, levels of lipids and lipoproteins (including cholesterol, triglycerides, HDL-C, LDL-C, ApoA-I, and ApoB) prior to the initial adjuvant chemotherapy were collected and recorded as lipid pre-chemotherapy (CT); levels measured at 2-3 weeks after the administration of last chemotherapy were recorded as lipid post-CT. For cohort 2, lipid and lipoprotein information was collected within 1 month after radical resection and half-year follow-up. The detection of lipids and lipoproteins was performed with early morning samples on an empty stomach and measured immediately by a Hitachi 7600-020 automatic biochemical analyzer (Hitachi High-Technologies, Tokyo, Japan) but were reviewed retrospectively.

### Treatment and follow-up

Whether the patients in this study received adjuvant chemotherapy after radical resection was based on pathological staging and clinical characteristics, as decided by the physicians in Sun Yat-sen University Cancer Center. Patients with stage III or high-risk stage II were recommended with fluoropyrimidine-based adjuvant chemotherapy except for those with serious comorbidities or poor anticipated life expectancy assessed by physicians. High-risk was defined as any of the following: pre-operative obstruction, perforation, T4 lesion, lymphovascular invasion, perineural invasion, close, indeterminate or positive margins, poorly differentiated histology or number of lymph nodes analyzed after surgery < 12. The fluoropyrimidine-based regimens included CapeOx (oxaliplatin and capecitabine, repeated every 3 weeks), FOLFOX (oxaliplatin, FU and LV, repeated every 2 weeks), capecitabine single agents (repeated every 3 weeks), 5-FU/LV (repeated every 2 weeks) and other regimens, including fluoropyrimidine. The addition of oxaliplatin was decided according to patients' tumor stage, age, risk factors and testing for mismatch repair proteins. Pre- or post-operative concurrent chemoradiotherapy for rectal cancer was permitted. Follow-up was performed every 3 months for the first 3 years and then every 6 months for 5 years in cohort 1 and cohort 2 patients. The date of relapse and death were confirmed by the hospital records or phone contact with the patient or their relatives. This study was approved by the institutional ethical review board of Sun Yat-sen University Cancer Center and was conducted in accordance with the Helsinki Declaration of the World Medical Association. Given the non-interventional retrospective design, informed consent was not required in this study.

### Statistical analysis

The pertinent patient characteristics and lipid changes were presented as descriptive statistics. Differences between lipid pre-CT and lipid post-CT were investigated using paired student *t*-test for each patient in cohort 1. Paired student *t*-test was also used to compare lipid levels in cohort 2. The predictive value of significantly changed serum lipids were analyzed for DFS and OS using the Kaplan-Meier method with log-rank test. DFS was defined as the date of treatment to the date of the first relapse at any site or death from any cause without relapse. OS was calculated from treatment to death from any cause. Χ^2^ tests were performed to evaluate the associations between variables of interest. The univariate analyzes were performed using Cox regression analysis (including age, sex, location of primary tumor, tumor grade, T-stage, N-stage, tumor size, pre-operative CEA, pre-operative CA19-9, BMI and all types of chemotherapy-related lipid alteration). Then, the variables that exhibited statistical significance (*P* < 0.05) in univariate analyses were tested in the multivariate analysis with forward stepwise Cox regression model. A stratified analysis was also performed to test whether the prognostic value of chemotherapy-related HDL-C elevation was associated with the oxaliplatin combined regimens. All of the statistical analyses were performed using SPSS software (version 22) with two-tailed tested. A *P* value of < 0.05 was considered statistically significance.

### Internal validation

A cross-validation re-sampling procedure was conducted to evaluate the robustness of the multivariate model and to validate the results [27, 28]. Briefly, one hundred less-powered simulation datasets were generated by randomly selecting 80% of the original dataset. For each simulation, the Cox multivariate models of DFS and OS were performed. The replication rate and the mean HR of each variable were also calculated to validation the consistency of the results.

## SUPPLEMENTARY MATERIAL TABLES


