# Supplementary material for: Predictive value of chemotherapy-related high-density lipoprotein cholesterol (HDL) elevation in patients with colorectal cancer receiving adjuvant chemotherapy: an exploratory analysis of 851 cases
Source: Oncotarget. 2016 Jun 17;7(35):57290–300. doi: 10.18632/oncotarget.10145 (PMC5302989; doi:10.18632/oncotarget.10145)
Supplement: Supplementary file 1 [file oncotarget-07-57290-s001.pdf]

Predictive value of chemotherapy-related high-density lipoprotein cholesterol (HDL) elevation in patients with colorectal cancer receiving adjuvant chemotherapy: an exploratory analysis of 851 cases

Supplementary Material

STable 1: Chemotherapy-related lipid alterations in different subgroups.

	Pre-CT	Post-CT	Difference[ɭ]	*P* value[ɸ]
**≤ 65 year**				
Cholesterol (mmol/L)	4.88±0.96	5.06±0.98	0.18±1.01	< **0.001**
Triglyceride (mmol/L)	1.39±0.89	1.69±0.99	0.29±1.10	< **0.001**
HDL-C (mmol/L)	1.14±0.30	1.37±0.38	0.22±0.36	< **0.001**
LDL-C (mmol/L)	3.09±0.85	2.95±0.89	-0.14±0.89	< **0.001**
ApoA-Ⅰ (g/L)	1.17±0.39	1.35±0.29	0.18±0.44	< **0.001**
ApoB (g/L)	0.97±0.53	0.91±0.25	-0.06±0.52	**0.006**
**＞ 65 year**				
Cholesterol (mmol/L)	5.08±1.05	5.23±1.10	0.16±1.15	0.172
Triglyceride (mmol/L)	1.50±0.84	1.77±1.09	0.28±0.98	**0.005**
HDL-C (mmol/L)	1.17±0.30	1.37±0.32	0.20±0.35	< **0.001**
LDL-C (mmol/L)	3.21±0.89	3.02±0.99	-0.19±1.04	0.067
ApoA-Ⅰ (g/L)	1.23±0.27	1.40±0.27	0.17±0.31	< **0.001**
ApoB (g/L)	1.01±0.26	0.98±0.30	-0.03±0.30	0.349
**Stage Ⅱ**				
Cholesterol (mmol/L)	4.87±0.93	5.02±0.92	0.15±0.96	**0.010**
Triglyceride (mmol/L)	1.40±0.81	1.78±1.07	0.37±0.97	< **0.001**
HDL-C (mmol/L)	1.15±0.30	1.37±0.36	0.22±0.34	< **0.001**
LDL-C (mmol/L)	3.02±0.83	2.87±0.85	-0.15±0.89	**0.005**
ApoA-Ⅰ (g/L)	1.19±0.46	1.37±0.26	0.18±0.49	< **0.001**
ApoB (g/L)	0.96±0.47	0.90±0.24	-0.06±0.46	**0.021**
**Stage Ⅲ**				
Cholesterol (mmol/L)	4.94±1.00	5.14±1.05	0.20±1.08	< **0.001**
Triglyceride (mmol/L)	1.41±0.93	1.64±0.95	0.23±1.16	< **0.001**
HDL-C (mmol/L)	1.14±0.31	1.37±0.37	0.23±0.37	< **0.001**
LDL-C (mmol/L)	3.17±0.87	3.03±0.94	-0.15±0.93	**0.002**
ApoA-Ⅰ (g/L)	1.17±0.29	1.35±0.30	0.18±0.37	< **0.001**
ApoB (g/L)	0.99±0.52	0.94±0.27	-0.05±0.52	0.060
**G1**				
Cholesterol (mmol/L)	4.74±1.00	5.01±1.04	0.27±1.06	**0.002**
Triglyceride (mmol/L)	1.35±0.60	1.77±0.90	0.42±0.89	< **0.001**
HDL-C (mmol/L)	1.10±0.30	1.35±0.36	0.25±0.37	< **0.001**
LDL-C (mmol/L)	2.99±0.86	2.87±0.92	-0.11±0.90	0.136

ApoA-Ⅰ (g/L)	1.18±0.60	1.33±0.30	0.15±0.65	**0.006**
ApoB (g/L)	0.93±0.32	0.90±0.26	-0.03±0.28	0.138
**G2-3**				
Cholesterol (mmol/L)	4.96±0.96	5.11±0.99	0.15±1.02	**0.001**
Triglyceride (mmol/L)	1.43±0.95	1.68±1.03	0.25±1.13	< **0.001**
HDL-C (mmol/L)	1.16±0.30	1.37±0.37	0.21±0.36	< **0.001**
LDL-C (mmol/L)	3.14±0.86	2.99±0.90	-0.16±0.91	< **0.001**
ApoA-Ⅰ (g/L)	1.18±0.27	1.36±0.28	0.19±0.33	< **0.001**
ApoB (g/L)	0.99±0.54	0.93±0.26	-0.06±0.54	**0.009**
**Male**				
Cholesterol (mmol/L)	4.84±0.98	5.05±1.01	0.21±1.05	< **0.001**
Triglyceride (mmol/L)	1.45±0.92	1.75±1.09	0.30±1.14	< **0.001**
HDL-C (mmol/L)	1.10±0.30	1.32±0.35	0.21±0.35	< **0.001**
LDL-C (mmol/L)	3.06±0.87	2.96±0.92	-0.10±0.92	**0.026**
ApoA-Ⅰ (g/L)	1.15±0.41	1.33±0.27	0.18±0.45	< **0.001**
ApoB (g/L)	0.98±0.60	0.92±0.27	-0.06±0.59	0.056
**Female**				
Cholesterol (mmol/L)	5.04±0.95	5.16±0.98	0.12±1.00	**0.06**
Triglyceride (mmol/L)	1.34±0.82	1.61±0.84	0.27±0.98	< **0.001**
HDL-C (mmol/L)	1.22±0.30	1.45±0.38	0.23±0.38	< **0.001**
LDL-C (mmol/L)	3.19±0.83	2.96±0.89	-0.22±0.90	< **0.001**
ApoA-Ⅰ (g/L)	1.22±0.30	1.40±0.30	0.18±0.37	< **0.001**
ApoB (g/L)	0.98±0.24	0.92±0.24	-0.06±0.25	< **0.001**
**＜ 24 kg/m^2**				
Cholesterol (mmol/L)	4.88±0.95	5.04±0.99	0.15±1.04	**0.002**
Triglyceride (mmol/L)	1.35±0.93	1.60±0.93	0.26±1.09	< **0.001**
HDL-C (mmol/L)	1.18±0.31	1.40±0.38	0.22±0.37	< **0.001**
LDL-C (mmol/L)	3.06±0.83	2.90±0.87	-0.16±0.90	< **0.001**
ApoA-Ⅰ (g/L)	1.19±0.26	1.36±0.29	0.17±0.33	< **0.001**
ApoB (g/L)	0.98±0.59	0.90±0.25	-0.07±0.57	**0.007**
**≥ 24 kg/m^2**				
Cholesterol (mmol/L)	4.97±1.02	5.20±1.01	0.22±1.00	**0.001**
Triglyceride (mmol/L)	1.53±0.76	1.89±1.11	0.36±1.07	< **0.001**
HDL-C (mmol/L)	1.07±0.27	1.30±0.32	0.23±0.33	< **0.001**
LDL-C (mmol/L)	3.20±0.91	3.08±0.95	-0.11±0.93	**0.068**
ApoA-Ⅰ (g/L)	1.16±0.53	1.34±0.27	0.18±0.57	< **0.001**
ApoB (g/L)	0.98±0.25	0.97±0.26	-0.02±0.27	0.298
**FOLFOX/CAPEOX**				
Cholesterol (mmol/L)	4.89±0.96	5.10±0.99	0.21±1.02	< **0.001**
Triglyceride (mmol/L)	1.41±0.92	1.67±0.97	0.26±1.08	< **0.001**
HDL-C (mmol/L)	1.14±0.29	1.38±0.38	0.24±0.36	< **0.001**
LDL-C (mmol/L)	3.10±0.85	2.96±0.88	-0.14±0.90	< **0.001**
ApoA-Ⅰ (g/L)	1.16±0.28	1.35±0.29	0.19±0.34	< **0.001**
ApoB (g/L)	0.98±0.53	0.92±0.25	-0.06±0.52	**0.007**

**5-Fu+LV/capecitabine**				
Cholesterol (mmol/L)	5.00±1.00	5.09±1.00	0.84±1.01	0.443
Triglyceride (mmol/L)	1.41±0.63	1.91±1.25	0.50±1.15	**< 0.001**
HDL-C (mmol/L)	1.22±0.33	1.33±0.31	0.11±0.34	**0.003**
LDL-C (mmol/L)	3.22±0.89	2.97±1.03	-0.25±0.97	**0.018**
ApoA-Ⅰ (g/L)	1.30±0.73	1.39±0.23	0.94±0.77	0.260
ApoB (g/L)	1.01±0.27	0.96±0.29	-0.05±0.29	**0.090**

HDL-C = high-density lipoprotein cholesterol, LDL-C = low-density lipoprotein cholesterol, ApoA-I = apolipoprotein A-I, ApoB = apolipoprotein B, CT = chemotherapy. Data are mean ± standard deviations. ʅDifference = $lipids_{post-CT}$ - $lipids_{pre-CT}$. ϕCompared with paired t-test.

STable 2: Prognostic factor definition according to internal validation.

Variables	Mean HR	SD	Replication Rate (%)
**Disease-free survival**			
Location of primary tumor	0.64	0.02	50
T-stage	2.15	0.33	95
N-stage	2.61	0.30	100
pre-operative CA19-9	1.65	0.11	50
HDL-C elevation	0.58	0.05	84
**Overall survival**			
Age	1.90	0.16	88
Gender	1.78	0.15	76
Location of primary tumor	0.55	0.05	96
N-stage	2.59	0.40	100
pre-operative CA19-9	1.75	0.13	67
HDL-C elevation*	0.56	0.06	86

HR = hazard ratio; SD = standard deviations. *Comparision between before and after chemotherapy.
